# Population-Based Inorganic Mercury Biomonitoring and the Identification of Skin Care Products as a Source of Exposure in New York City

**DOI:** 10.1289/ehp.1002396

**Published:** 2010-10-05

**Authors:** Wendy McKelvey, Nancy Jeffery, Nancy Clark, Daniel Kass, Patrick J. Parsons

**Affiliations:** 1 Division of Environmental Health, New York City Department of Health and Mental Hygiene, New York, New York, USA; 2 Laboratory of Inorganic and Nuclear Chemistry, Wadsworth Center, New York State Department of Health, Albany, New York, USA; 3 Department of Environmental Health Sciences, School of Public Health, University at Albany, State University of New York, Albany, New York, USA

**Keywords:** biomonitoring, inorganic mercury poisoning, mercury, National Health and Nutrition Examination Survey, New York City, NYC HANES, skin care, skin-lightening creams, urine

## Abstract

**Background:**

Mercury is a toxic metal that has been used for centuries as a constituent of medicines and other items.

**Objective:**

We assessed exposure to inorganic mercury in the adult population of New York City (NYC).

**Methods:**

We measured mercury concentrations in spot urine specimens from a representative sample of 1,840 adult New Yorkers in the 2004 NYC Health and Nutrition Examination Survey. Cases with urine concentrations ≥ 20 μg/L were followed up with a telephone or in-person interview that asked about potential sources of exposure, including ritualistic/cultural practices, skin care products, mercury spills, herbal medicine products, and fish.

**Results:**

Geometric mean urine mercury concentration in NYC was higher for Caribbean-born blacks [1.39 μg/L; 95% confidence interval (CI), 1.14–1.70] and Dominicans (1.04 μg/L; 95% CI, 0.82–1.33) than for non-Hispanic whites (0.67 μg/L; 95% CI, 0.60–0.75) or other racial/ethnic groups. It was also higher among those who reported at least 20 fish meals in the past 30 days (1.02 μg/L; 95% CI, 0.83–1.25) than among those who reported no fish meals (0.50 μg/L; 95% CI, 0.41–0.61). We observed the highest 95th percentile of exposure (21.18 μg/L; 95% CI, 7.25–51.29) among Dominican women. Mercury-containing skin-lightening creams were a source of exposure among those most highly exposed, and we subsequently identified 12 imported products containing illegal levels of mercury in NYC stores.

**Conclusion:**

Population-based biomonitoring identified a previously unrecognized source of exposure to inorganic mercury among NYC residents. In response, the NYC Health Department embargoed products and notified store owners and the public that skin-lightening creams and other skin care products that contain mercury are dangerous and illegal. Although exposure to inorganic mercury is not a widespread problem in NYC, users of these products may be at risk of health effects from exposure.

Mercury is a toxic metal that has been used by humans for centuries as a constituent of medicines, scientific instruments, fungicides, and other items, in addition to being used in many industrial processes (Goldwater 1972). It is most recognizable in its elemental (metallic) state (Hg^0^) as a silvery liquid at room temperature. However, in its most common oxidation state (Hg^2+^), it can form a wide range of compounds.

In the United States, people are exposed to mercury most frequently by eating fish in which the organic compound methylmercury (CH_3_Hg^+^) has accumulated. However, exposure to inorganic forms of mercury can also occur such as during inhalation of vapors from accidental spills, in workplace activities (e.g., the manufacture of chloralkali compounds and mercury-containing devices), from dental amalgams, or from ritualistic or cultural practices that involve elemental mercury [Agency for Toxicological Substances and Disease Registry (ATSDR) 1999]. Approximately 80% of elemental mercury vapor is absorbed by the human body through inhalation, in contrast with ingestion or dermal exposure to elemental mercury, which rarely results in toxicity [International Program on Chemical Safety (IPCS) 1991]. Once absorbed, the dissolved vapor readily crosses the placenta and the blood–brain barrier, where it can be harmful to the developing nervous system or interfere with neurological function. The kidney is also a principal site of toxicity (Clarkson et al. 2003).

In New York City (NYC), the relative importance of exposure to elemental mercury has been debated [Newby et al. 2006; U.S. Environmental Protection Agency (EPA) 2002; Wendroff 1997]. Certain Afro-Caribbean and Latin American traditions, including Santeria, Espiritismo, Palo Mayombe, and Voodoo have been known to practice rituals that involve sprinkling mercury around a home, wearing it in an ampule or amulet, burning it in a candle, or mixing it with perfume (Riley et al. 2001). These practices are believed to ward off evil, provide protection, and bring good luck (U.S. EPA 2002). They can also result in mercury volatilization and human exposure via inhalation, especially when large amounts, high temperatures, and frequent handling are involved (Riley et al. 2001).

Ingestion of traditional medicine products and the topical application of skin-lightening creams and other skin care products that contain mercury are also known sources of exposure to inorganic mercury. Small amounts of ingested inorganic mercury compounds may be absorbed by the gastrointestinal tract (up to 10%, although the amount may be greater in young children), but only a fraction of this amount is likely to cross the blood–brain barrier (IPCS 1991). The kidney is the primary target of toxicity for ingested inorganic mercury compounds (IPCS 1976). Topically applied mercury compounds are more readily absorbed and are also most likely to accumulate in and cause damage to the kidneys, although high levels of exposure may also affect the nervous system (IPCS 1991; Kern et al. 1991). Topical exposures have also been known to cause acrodynia in children. This condition manifests with cardiovascular, dermal, and neurological symptoms such as irritability, photophobia, pink discoloration of the hands and feet, and polyneuritis (Boyd et al. 2000).

In 2004, NYC conducted the first local health and nutrition examination survey (HANES) in the United States, using a representative sample of adults. The survey was modeled after the National Health and Nutrition Examination Survey (NHANES)[Centers for Disease Control and Prevention (CDC) 2010] and included a biomonitoring component that measured total mercury concentration in blood and in urine. The latter is typically used to assess exposure to inorganic mercury, because virtually all mercury in urine is inorganic (ATSDR 1999; Carrier et al. 2001). Local biomonitoring was conducted under the premise that the relative importance of population exposures and sources can vary according to cultural, behavioral, and demographic characteristics of an area.

Using the results from the NYC HANES, we have described the distribution and determinants of exposure to inorganic mercury in NYC adults, as well as the NYC Department of Health and Mental Hygiene (DOHMH) response to the findings.

## Materials and Methods

### Sample selection

The NYC HANES was a population-based, cross-sectional survey that represented the civilian, noninstitutionalized adult (≥ 20 years of age) population residing in the five boroughs of NYC (Bronx, Brooklyn, Manhattan, Queens, and Staten Island). The survey was conducted between June and December 2004. Participants were recruited into the study using a three-stage cluster- sampling design. The stages of sample selection were *a*) selection of census blocks, or groups of blocks; *b*) enumeration and random selection of households within selected areas; and *c*) random selection of study participants within households. The target sample size was 2,000.

### Data collection

Individuals who were selected to participate in the study were invited to any of four clinic sites in the boroughs of Manhattan, Brooklyn, the Bronx, or Queens to be interviewed and examined (including blood and urine collection). Using a face-to-face, computer-assisted personal interview, study participants were asked their age, sex, race/ethnicity [white; black or African American; Asian/Hawaiian/Pacific Islander (referred to here as Asian); Native American/Alaskan Native or other; and whether they considered themselves to be Hispanic or Latino], education, income, place of birth, and length of time in the United States. Participants who identified themselves as Hispanic were asked about their country of origin. All participants were asked about the number of fish or shellfish meals they had eaten in the past 30 days. We translated the survey instrument into Spanish; interviews in other languages were conducted by an NYC DOHMH staff member, by a participant’s family member, or by a telephone translation service (Language Line, Monterey, CA).

Spot urine specimens were collected using supplies certified for trace metal measurements by the Wadsworth Center’s Laboratory of Inorganic and Nuclear Chemistry (clinical trace elements section) at the New York State Department of Health (NYS DOH) in Albany, NY. Aliquots of urine preserved with sulfamic acid and Triton-X 100 (Sigma-Aldrich Corp., St. Louis, MO) to prevent mercury loss were shipped on dry ice in Nalgene cryovials (Thermo Fisher Scientific, Rochester, NY) to the Wadsworth Center laboratory at the New York State Department of Health. Specimens were stored at −80 °C (Thermo Scientific, Asheville, NC) until they could be analyzed. The Wadsworth Center is certified under the federal Clinical Laboratory Improvements Amendments of 1988 (CLIA; Centers for Medicaid and Medicare Services 2010) and holds a clinical laboratory permit for measuring trace elements from NYS DOH.

The NYC HANES protocol was approved by the NYC DOHMH and the NYS DOH institutional review boards. Study participants provided written, informed consent, and those who provided interview and laboratory data were remunerated $100. Information on data collection and protocols, as well as a detailed description of the study design, have been published elsewhere (Thorpe et al. 2006).

### Laboratory methods

Total mercury concentration in urine was determined using a method optimized for a PerkinElmer Sciex (Shelton, CT) ELAN DRC II inductively coupled plasma mass spectrometer (ICP-MS) (Parsons et al. 2005). The Wadsworth Center laboratory calibrated the instrument using inorganic mercury standards traceable to the National Institute of Standards and Technology (NIST; Gaithersburg, MD). Five different concentrations of internal quality control (IQC) materials that covered the range of exposure expected in the U.S. population were analyzed at the beginning and end of each batch of specimens and throughout each analytical run. The coefficient of variation (a measure of reproducibility) varied from 4.5% at 3.8 ug/L to 6.1% at 38 ug/L. NIST Standard Reference Material (SRM) 2670a, Toxic Metals in Freeze-Dried Urine (NIST 2003), was analyzed periodically throughout the study. We reanalyzed specimens that had mercury concentrations of 20 ug/L and above. We also randomly selected 2.5% of all specimens for a repeat analysis. The limit of detection was 0.11 μg/L. Quality control performance for this method has been described elsewhere (Minnich et al. 2008).

The analytical performance for the ICP-MS urine mercury method has also been assessed periodically by external quality assessment schemes (EQAS) operated by the Institut national de santé publique du Québec, Le centre de toxicology du Québec; the German EQAS, University Erlangen-Nuremberg, Germany; the United Kingdom National EQAS for Trace Elements; and the Programa Interlaboratorios de Control de Calidad de Metales en Orina (PICC- Met U), Spain, and has been found to be satisfactory. In addition, the Wadsworth Center’s Laboratory of Inorganic and Nuclear Chemistry organizes and participates in the NYS DOH proficiency testing scheme for urine mercury.

Mercury concentrations are presented both uncorrected (micrograms per liter) and corrected for creatinine (micrograms per gram). The National Center for Environmental Health at the CDC measured creatinine concentrations using the Roche Creatinine Plus Assay and a Roche Hitachi Automatic Analyzer, Model 912 (Hitachi, Inc., Tokyo, Japan). Creatinine excretion is often used to correct for (or normalize) the variable urine dilutions in spot urine samples, which are collected at random times throughout the day. Urinary creatinine values were unavailable for 17 of the 1,840 study participants for whom urine was collected: 6 specimen results were excluded because the measured values were ≤ 0.3 mg/dL, and 11 were unavailable because the urine quantity was insufficient or the sample was lost or damaged during transport. The creatinine assays were performed in 2005 on a subset of the urine specimens and completed for the remaining specimens in 2009.

### Statistical analysis

We applied sample weights to all estimates to adjust for differential selection probabilities and survey nonresponse. Weights were poststratified to reflect the age, sex, race/ethnicity, and borough of residence of the NYC population (U.S. Census Bureau 2006). We conducted statistical analyses using SAS (version 9.1; SAS Institute Inc., Cary, NC), and SUDAAN 10 (Research Triangle Institute, Research Triangle Park, NC) to account for the complex sampling design. Mercury levels below the limit of detection (0.11 μg/L) were assigned a value equal to the limit of detection divided by the square root of two.

We calculated crude population-weighted geometric means for urine mercury concentrations by taking the antilog of the mean of the natural log-transformed values. Upon visual inspection, logging the values made a substantial improvement toward the approximation of a normal distribution. We used *t*-tests to compare categorical estimates and considered a difference to be statistically significant at *p* < 0.05. We used the method of Korn and Graubard (1998) to estimate the population-weighted 95th percentiles and their 95% confidence intervals (CIs).

We fitted multiple linear regressions of the natural log-transformed urine mercury concentrations on the predictor variables, excluding persons categorized as Native American or Non-Hispanic Other because of insufficient sample size, and those with missing covariate data. We present the exponentiated model coefficients, which can be interpreted as the proportional change in the arithmetic mean associated with each level of the predictor, relative to a referent level, after adjusting for the other predictors in the model. We included creatinine concentration in all models, rather than correcting the urine mercury concentrations directly (Barr et al. 2005). We also regressed the log-urine mercury on log-blood mercury concentrations to measure the strength of association between these two biomarkers of exposure [measurements of total mercury in blood have been described elsewhere (McKelvey et al. 2007)]; we assessed correlations using the Pearson correlation coefficient. We considered a result to be statistically significant if the 95% CI did not include 1.0.

### Follow-up of participants with elevated levels of mercury and identification of exposure sources

The study team followed up with those participants who had urine mercury levels ≥ 20 μg/L (NYS reportable level; NYS DOH 2010) by administering a telephone interview or an in-person interview that asked about potential sources of exposure (e.g., ritualistic and cultural practices, skin care products, metallic mercury spills, herbal medicine products, fish). When a participant reported using a potentially toxic product, we collected information on the product, including its name, where it was obtained, and how it was used. We provided education to all participants with elevated mercury levels on the potential health effects of mercury and how to reduce exposure.

## Results

Of the 3,634 selected, eligible survey participants, 1,999 persons completed the interview and at least one component of the examination, which yielded an overall survey response rate of 55%. Measurements of urine mercury concentrations were available for 1,840 participants (92%), yielding a response rate of 51% for this analysis. There was no intentional oversampling of particular demographic subgroups; we used poststratification survey weighting to ensure that the age, sex, race/ethnicity, and the distributuion of the participants residing in the study boroughs were the same as those of the general NYC population (U.S. Census Bureau 2006).

The geometric mean urine mercury concentration among NYC adults was 0.73 μg/L (95% CI, 0.69–0.79), which is similar to the creatinine-corrected mean of 0.69 μg/g (95% CI, 0.65–0.73) (Tables 1 and 2). A total of 156 samples (9%) were below the limit of detection (assigned a value of 0.078 μg/L for subsequent analyses); for the remaining 1,684 samples, the mean urine mercury concentrations ranged from 0.11 to 95 μg/L.

In Tables 1 and 2, we present uncorrected and creatinine-corrected urine mercury concentrations stratified by demographic characteristics and fish consumption. The results of the multiple regression modeling in Table 2 estimate the proportionate change in mercury levels across categories of a predictor when holding other model covariates constant. Patterns of change are similar to those of the geometric mean estimates across category levels. Women had higher geometric means than did men in both uncorrected (*p* = 0.07) and creatinine-corrected analyses (*p* < 0.01), with the corrected analyses showing stronger evidence for a difference. Urine mercury levels increased with age until the fifth or sixth decade, at which point they dropped (Table 2). Fish consumption showed the strongest association with urine mercury levels, based on the proportionate increase in urine mercury concentration among those who consumed fish or shellfish ≥ 20 times in the past 30 days, compared with those who never ate fish or shellfish (Table 2). Urine mercury levels across all categories of fish consumption were statistically significantly different (*p* < 0.05) in both uncorrected and creatinine-corrected analyses for all two-way comparisons except for the comparisons between the two highest categories.

Thirteen individuals (all women) had urine mercury concentrations that equaled or exceeded the NYS reportable level of 20 μg/L (weighted NYC population estimate = 26,690). All of the study participants with elevated levels were either Hispanic (*n* = 11) or black (*n* = 2) between the ages of 21 and 51 years, and all but one black woman were born outside the United States. Of the 11 Hispanic women, 10 were born in the Dominican Republic; 4 Dominican women had mercury levels > 50 μg/L. The 97.5th percentile for urine mercury concentration overall was 6.13 μg/L (5.25 μg/g creatinine).

During follow-up interviews with study participants who had urine mercury levels ≥ 20 μg/L, we identified mercury-containing skin-lightening creams as the primary exposure source in 9 of 13 cases; we were unable to identify a source for 4 individuals. We identified and confiscated Recetas de la Farmacia Normal – Crema Blanqueadora during a visit to the home of the study participant who had the highest mercury level (95 μg/L). This woman had reportedly brought this product into NYC from the Dominican Republic. It was tested at a commercial laboratory, certified by the NYS DOH Wadsworth Center Environmental Laboratory Accreditation Program, and found to contain a mercury concentration of 6,190 ppm (0.62% mercury by weight).

Because we documented the use of mercury-containing skin-lightening creams by Dominicans and other Hispanic racial/ethnic groups and by Caribbean-born blacks, we looked specifically at urine mercury concentrations among participants in these groups (Tables 1 and 2). The 97 study participants who were categorized as non-Hispanic, Caribbean-born black were born in Antigua and Barbuda, Bahamas, Barbados, Belize, Bermuda, Cuba, Grenada, Haiti, Saint Kitts and Nevis, Saint Lucia, Saint Vincent and the Grenadines, Trinidad and Tobago, or the United States or British Virgin Islands. The 95th percentile of urine mercury levels among Dominicans was highest (21.2 μg/L) compared with all other race/ethnicity groups, exceeding the NYS reportable level (20 μg/L). Caribbean-born blacks and Dominicans had the highest geometric mean urine mercury levels in both creatinine-corrected and uncorrected analyses (*p* < 0.01 for each, compared with study participants who were neither Caribbean-born black nor Dominican).

We observed a statistically significant positive association (*p* < 0.01) between urine and blood mercury levels when regressing the urine mercury on blood mercury concentrations. Although the correlation was not high (Pearson *r*^2^ = 31%), we found that participants with the highest urine mercury levels (≥ 10 μg/L) had a geometric mean blood mercury concentration of 8.79 μg/L, whereas those with urine mercury levels less than 10 μg/L had a geometric mean of 2.70 μg/L.

### Public health actions

Upon discovering that illegal mercury-containing skin-lightening creams were being used in NYC, DOHMH sent out press releases in English and Spanish urging people to stop using these products immediately and issued an electronic health alert to health care providers. The public was asked to report the names of any mercury-containing skin-lightening creams they had purchased or used and the stores that were selling them to the NYC Poison Control Center (PCC). The PCC received 139 calls within a 2-week period in early 2005.

The DOHMH also mailed bilingual press releases and consumer warning signs to 270 non–chain pharmacies, discount and beauty supply stores, and botanicas located in neighborhoods with large Dominican populations or in ZIP codes where members of the public had purchased products (NYC DOHMH 2005). A team from DOHMH subsequently visited these stores and similar stores nearby to verify that products with mercury listed as an ingredient had been removed from the shelves. The Commissioner of Health issued orders to embargo or seize remaining products, and store owners were required to provide the names of distributors and to post warning signs in their stores.

Ultimately, 17 different products were embargoed or seized from 22 stores and subsequently tested for mercury. Of the 12 products that contained mercury concentrations that exceeded the U.S. Food and Drug Administration (FDA) allowable level of 1 ppm, eight were skin-lightening or beauty creams; nine were labeled as manufactured in the Dominican Republic, and seven listed mercury as an ingredient on the product label (Table 3). Four of the 12 products were antiseptic soaps or creams that were labeled as manufactured in the Dominican Republic or the European Economic Community. Products were sent to the U.S. FDA Region 2 (Queens, NY) laboratory for analysis. Store investigations identified six distributors located in New Jersey who had been supplying products to NYC stores.

## Discussion

NYC conducted the first local HANES in the United States under the premise that results from national public health surveillance are likely to vary according to regional differences in cultural, behavioral, and demographic composition of the population. Local surveillance provides more relevant information for targeting resources and outreach and offers an opportunity to assess the impact of local exposure sources on the population. We demonstrated the unique value of local data by identifying contaminated skin care products as a previously unrecognized source of exposure to inorganic mercury in NYC.

Population-based biomonitoring confirmed that exposure to inorganic mercury from use of mercury-containing skin-lightening creams had occurred in Dominican and possibly Caribbean groups in NYC. This exposure source has been described in previous investigations of poisonings due to use of these products in California, Texas, and New Mexico (CDC 1996; Weldon et al. 2000). The NYC HANES did not query participants about their use of mercury- containing products, so we are unable to report prevalence of use in NYC. However, in a 1997 survey of Hispanic communities on the Texas–Mexico border, 5% of households reported at least one person who had used a mercury-containing skin-lightening cream in the past year (Weldon et al. 2000). In contrast to previous investigations, the relatively low urine mercury levels among Mexican New Yorkers did not suggest that use of mercury-containing creams was common in this group. However, the number of Mexicans surveyed was small (*n* = 78).

In the United States, it is illegal to sell any skin care product that contains more than 1 μg/g (ppm) mercury, with the exception of eye-area cosmetics or drops, which may contain up to 65 μg/g. However mercury- containing creams and soaps remain unregulated and available in other countries (IPCS 1991). In 2003, the U.S. FDA issued import alerts on Manning Beauty cream (from Mexico), and Dermaline, Miss Key, and Santa Crema creams (from the Dominican Republic) (U.S. FDA 2003). Three of these products and other similar ones were found in NYC stores in 2005 (Table 3). The products tested by the NYC DOHMH contained mercury concentrations up to 41,600 μg/g, or about 4.2% by weight (Table 3). We learned from store owners that products were being distributed by wholesalers in New Jersey, which led the New Jersey Department of Health and Senior Services to launch an investigation shortly thereafter to embargo products at the wholesale level.

After determining that many products seized from NYC shelves were manufactured in the Dominican Republic, the NYC DOHMH met with the Dominican Consulate. We also communicated our findings to the Pan American Health Organization. Several weeks later, their local office reported to us that the Dominican Secretary of Health had notified all laboratories to stop manufacturing mercury-containing skin care products. Enforcement of the notification will require continued political and regulatory support, but we believe that studies documenting the potentially toxic exposure levels that may occur from using contaminated products can be used to leverage such support.

We were unable to document exposure to inorganic mercury through cultural or ritualistic use of mercury in NYC, among the NYC HANES participants with elevated urine mercury levels. Few studies describe mercury exposure resulting from ritualistic practices, but some have suggested that such practices may not necessarily result in high or prolonged exposures (Riley et al. 2001; Singhvi 2005). Nonetheless, many jurisdictions, including NYS, have passed legislation to limit the sale of elemental mercury to reduce the potential for exposure and mismanagement (NYS Department of Environmental Conservation 2010).

Overall, our results suggest that exposure to inorganic mercury in NYC adults is slightly higher than national levels for men and more so for women, based on a comparison of NHANES 2003–2004 estimates (Table 4) (CDC 2009). Germany is one of the few countries that has also conducted population-based urine mercury biomonitoring. The creatinine-corrected geometric mean for NYC adults was 0.69 (95% CI, 0.65–0.73) compared with 0.34 (95% CI, 0.33–0.35) μg/g creatinine from the 1998 German Environmental Surveys (Becker et al. 2003). However, the overall NYC geometric mean is not as high as the geometric mean among German study participants who had mercury amalgams on at least eight teeth (0.89 μg/g; 95% CI, 0.82–0.97). In a nonoccupational setting, the presence of mercury amalgams in tooth fillings is a strong predictor of urine mercury levels (Akesson et al. 1991; Kingman et al. 1998). We did not survey for the presence of amalgams, so we are unable to assess the extent to which mercury amalgam fillings influence urine mercury levels in NYC adults.

However, we were able to corroborate higher urine mercury levels in individuals who consumed fish most frequently, similar to other studies (Apostoli et al. 2002; Levy et al. 2004). These observations challenge the currently held notion that methylmercury in fish is not likely to impact mercury exposure or accumulation in the kidney (Clarkson and Magos 2006). Virtually all mercury present in urine is of the inorganic form, whereas mercury in fish is predominantly methylated (ATSDR 1999; Carrier et al. 2001; IPCS 1976). One explanation for the observed association between fish consumption and urine mercury levels is demethylation of methylmercury *in vivo*, with subsequent elimination via the kidneys (IPCS 1990). The fish and urine mercury association may also explain part of the blood and urine mercury association we observed, as fish consumption is also positively associated with blood mercury levels (McKelvey et al. 2007).

The NYC HANES conducted in 2004 opted to conduct both blood and urine mercury biomonitoring. This choice was fortunate, as blood mercury biomonitoring alone might not have led to the discovery of skin-lightening creams as an exposure source. Blood mercury levels reported from the NYC HANES identified fish consumption as a major source of exposure. Hispanics had relatively low levels of fish consumption and therefore were not identified as a subgroup with higher risk of exposure to mercury (McKelvey et al. 2007). An alternative to biomonitoring for mercury exposure in both urine and blood, for the purpose of identifying different exposure sources, would be to use a speciation method that distinguishes between methylmercury and inorganic mercury in blood.

Interpretation of our findings has some limitations. Although the NYC HANES sample selection was designed to be representative of the NYC adult population, we cannot rule out the presence of bias in our population estimates, because the overall response rate was 51%. However, we have attempted to correct for differences between responders and nonresponders using sample weights that take into account census block (or block group) characteristics such as education, income, foreign language spoken at home, racial/ethnic composition, household size, and home ownership. Weights were further poststratified and calibrated so that all NYC HANES estimates are consistent with the cross-tabulated age, race/ethnicity, sex, and the population sizes of the borough of residence, as estimated by the U.S. Census Bureau (2006). We also noted that the national HANES interview and examination response rate for a population of similar age in the NYC area in 2004 was only slightly higher [58% (Porter K, NHANES program, personal communication)] than the 55% response rate in the NYC HANES. Response rates for the urine collection component of the examination were slightly lower in both the U.S. and NYC surveys. Nonetheless, it remains possible that we did not achieve representativeness within the subgroups considered for our weighting scheme, which could produce inaccuracy in our population estimates.

Laboratory methods for measuring chemical exposures have become increasingly sensitive, so detecting low levels of mercury in the urine of an adult does not necessarily imply a health risk. Cohort studies of children who have urine mercury levels similar to the geometric means we report here have not revealed adverse renal or neuropsychological effects (Bellinger et al. 2006; DeRouen et al. 2006). However, some occupational studies have documented harmful effects to the kidneys and nervous system of workers who have urine mercury levels in the range of 20–50 μg/g creatinine (IPCS 1991). NYC HANES results suggest that almost 27,000 New Yorkers (estimate based on the subset of 13 women with such levels) are at risk of exposure at this level.

## Conclusion

Population-based biomonitoring proved to be a valuable tool for identifying a previously unrecognized source of exposure to mercury in NYC. The NYC Health Department responded to this finding by embargoing illegal products and notifying store owners and the public that mercury-containing skin- lightening creams and other skin care products are dangerous and illegal. Although exposure to inorganic mercury is not a widespread problem in NYC, a number of New Yorkers who use these products may be at risk of adverse health effects.

## Figures and Tables

**Table 1:** Urine mercury concentrations, geometric means, and 95th percentiles for NYC adults.

Characteristics	*n*^a^	Population-weighted geometric mean urine mercury [μg/L (95% CI)]	Population-weighted 95th percentile urine mercury [μg/L (95% CI)]
Total	1,840	0.73 (0.69–0.79)	4.35 (3.95–4.73)

Sex			

Male	766	0.69 (0.63–0.76)	3.77 (3.50–4.36)
Female	1,074	0.77 (0.71–0.85)	4.76 (4.20–5.76)

Age (years)			

20–29	490	0.66 (0.59–0.74)	4.52 (3.71–5.76)
30–39	425	0.80 (0.70–0.92)	4.75 (4.01–5.55)
40–49	404	0.93 (0.82–1.06)	5.05 (4.12–8.32)
50–59	283	0.80 (0.69–0.94)	4.48 (3.72–6.3)
> 60	238	0.56 (0.48–0.64)	2.70 (2.13–3.04)

Race/ethnicity^b^			

White, non-Hispanic	538	0.67 (0.60–0.75)	3.84 (3.50–4.46)
Black, non-Hispanic	398	0.89 (0.78–1.00)	4.40 (3.62–5.31)
Black, Caribbean-born non-Hispanic	97	1.39 (1.14–1.70)	4.46 (3.61–10.52)
Asian, non-Hispanic	235	0.63 (0.51–0.78)	3.90 (3.09–4.42)
Hispanic	638	0.77 (0.69–0.87)	5.05 (4.31–6.95)
Foreign-born Dominican	149	1.04 (0.82–1.33)	21.18 (7.25–51.29)
Puerto Rican^c^	178	0.79 (0.67–0.94)	3.52 (2.63–6.36)
Foreign-born Mexican	78	0.33 (0.24–0.46)	1.90 (1.11–2.74)

Family income ($US)			

< 20,000	618	0.66 (0.58–0.74)	5.05 (3.88–6.99)
20,000–49,999	579	0.76 (0.69–0.85)	4.20 (3.72–4.88)
50,000–74,999	259	0.70 (0.59–0.83)	3.70 (3.22–4.14)
≥ 75,000	306	0.92 (0.81–1.05)	4.72 (3.5–6.15)

Education			

< High school diploma	527	0.64 (0.56–0.73)	3.99 (3.37–5.11)
≥ High school diploma	1,305	0.77 (0.71–0.83)	4.40 (4.02–4.76)

Place of birth			

Continental United States	817	0.70 (0.65–0.76)	3.89 (3.55–4.54)
Outside continental United States^d^	1,017	0.77 (0.69–0.86)	4.42 (4.01–5.35)

Fish or shellfish consumption (number of times in past 30 days)			

Never	215	0.50 (0.41–0.61)	3.52 (2.33–6.36)
Up to 9	1,233	0.71 (0.66–0.77)	4.12 (3.72–4.48)
10–19	259	0.92 (0.79–1.07)	5.34 (4.26–9.46)
≥ 20	116	1.02 (0.83–1.25)	3.90 (3.56–5.71)

a Number of participants does not equal 1,840 because of missing data.

b Excludes 27 participants who classified themselves as “other.”

c Puerto Ricans were not considered foreign born.

d Includes Puerto Rico and U.S. Virgin Islands.

**Table 2:** Urine mercury concentrations corrected for creatinine concentrations, geometric means, adjusted proportional change in means, and 95th percentiles for NYC adults.

Characteristics	*n*^b^	Population-weighted geometric mean urine mercury [μg/g (95% CI)]	Adjusted proportional change in mean urine mercury^a^ [μg/g (95% CI)]	Population-weighted 95th percentile urine mercury [μg/g (95% CI)]
Total	1,823	0.69 (0.65–0.73)		3.75 (3.27–4.36)

Sex				

Male	758	0.53 (0.49–0.58)	1.00 (reference)	2.62 (2.20–3.06)
Female	1,065	0.86 (0.80–0.93)	1.44 (1.29–1.62)	4.73 (4.21–5.40)

Age (years)				

20–29	486	0.53 (0.47–0.59)	1.00 (reference)	3.54 (2.62–4.79)
30–39	422	0.69 (0.62–0.77)	1.23 (1.06–1.43)	3.24 (2.61–4.27)
40–49	399	0.89 (0.79–1.01)	1.50 (1.30–1.74)	5.25 (3.84–7.40)
50–59	280	0.88 (0.77–1.00)	1.43 (1.21–1.69)	4.52 (3.12–6.32)
> 60	236	0.58 (0.51–0.66)	1.12 (0.94–1.34)	2.97 (2.12–3.26)

Race/ethnicity^c^				

White, non-Hispanic	532	0.72 (0.64–0.80)	1.00 (reference)	3.58 (3.12–4.40)
Black, non-Hispanic	395	0.63 (0.56–0.70)	0.83 (0.71–0.97)	3.01 (2.58–3.67)
Black, Caribbean-born non-Hispanic	97	1.13 (0.95–1.34)	1.45 (1.15–1.82)	4.44 (3.01–6.98)
Asian, non-Hispanic	233	0.77 (0.63–0.93)	0.93 (0.74–1.17)	5.40 (2.72–9.49)
Hispanic	632	0.68 (0.61–0.76)	1.02 (0.87–1.20)	4.33 (3.35–5.69)
Foreign-born Dominican	147	1.00 (0.8–1.25)	1.53 (1.17–2.01)	26.52 (4.87–36.89)
Puerto Rican^d^	175	0.58 (0.49–0.69)		2.59 (1.64–4.35)
Foreign-born Mexican	78	0.31 (0.24–0.39)		1.82 (0.91–2.48)

Family income ($US)				

< 20,000	610	0.63 (0.56–0.70)	1.00 (reference)	4.27 (3.16–5.12)
20,000–49,999	575	0.68 (0.62–0.75)	1.12 (0.98–1.28)	3.26 (2.79–4.51)
50,000–74,999	259	0.66 (0.58–0.74)	1.07 (0.90–1.26)	3.35 (2.40–4.64)
≥ 75,000	301	0.88 (0.77–1.01)	1.39 (1.18–1.62)	4.21 (3.23–5.40)

Education				

< High school diploma	521	0.61 (0.54–0.69)	1.00 (reference)	3.35 (2.72–4.87)
≥ High school diploma	1,294	0.72 (0.67–0.77)	1.09 (0.96–1.24)	3.82 (3.26–4.40)

Place of birth				

Continental United States	810	0.63 (0.58–0.68)	1.00 (reference)	3.89 (2.95–4.30)
Outside continental United States^e^	1,006	0.76 (0.69–0.83)	1.14 (0.98–1.31)	4.30 (3.45–5.00)

Fish or shellfish consumption (number of times in past 30 days)				

Never	214	0.44 (0.36–0.54)	1.00 (reference)	3.19 (2.32–5.8)
Up to 9	1,221	0.68 (0.63–0.73)	1.43 (1.16–1.75)	3.41 (3.06–3.83)
10–19	255	0.83 (0.71–0.97)	1.74 (1.32–2.29)	4.38 (3.16–5.64)
≥ 20	116	1.03 (0.85–1.23)	2.23 (1.69–2.94)	4.79 (3.12–5.8)

a The exponentiated β coefficient from a log-linear multiple regression that includes all covariates in the table; for the adjusted analysis, *n* = 1,715 after excluding participants with missing covariate data.

b Number of participants does not equal 1,823 because of missing data.

c Excludes 27 participants who classified themselves as “other.”

d Puerto Ricans were not considered foreign born.

e Includes Puerto Rico and U.S. Virgin Islands.

**Table 3:** Results of mercury testing for products obtained from nonchain pharmacies, health and beauty supply stores, discount stores, and botanicas in NYC, 2005.

Product name	Place of manufacture	Active ingredient listed	Mercury content (μg/g)	Type of store(s) selling product
Skin-lightening creams				

Recetas de la Farmacia Normal–Crema Blanqueadora	Dominican Republic	Ammoniated mercury	6,190–41,600	Brought into country by study participant/Botanica
Dermaline Skin Cream	Dominican Republic	Amide chloride of mercury	21,100	Beauty supply
Magia Blanca de Michelle Marie Crema Blanqueadora	Unknown	No mercury listed	18,500	Pharmacy
Dermaline Skin Whitening Cream	Dominican Republic	No mercury listed	13,600	Beauty supply
Miss Key Crema Blanqueadora	Dominican Republic	Amide chloride of mercury	9,100	Pharmacy/beauty supply
Crema Santa	Dominican Republic	Mercury oxide	6,200	Pharmacy/discount/beauty supply
Deluxe Nadinola Bleaching Cream	Jamaica	3% Ammoniated mercury	3.47	Beauty supply
Dermaline Beauty Cream	Dominican Republic	No mercury listed	3.37	Pharmacy

Germicidal soaps/creams/balms				

Germicida 200 (soap)	European Economic Community	No mercury listed	4,770	Botanica
Crema Santa Germicida	Dominican Republic	No mercury listed	4,700	Pharmacy
Pomada Salva-Vida (balm)	Dominican Republic	Percl. mercurio: 10%	438	Botanica
Jabon Germicida Contifarma (soap)	Dominican Republic	1% Mercury iodine	204	Pharmacy/beauty supply

**Table 4:** Geometric means and 95th percentiles for adults ≥ 20 years of age who resided in NYC compared with the United States, NYC HANES 2004 and NHANES 2003–2004.

Survey	*n*	Crude-weighted geometric mean urine mercury [μg/L (95% CI)]	Crude-weighted 95th percentile urine mercury [μg/L (95% CI)]
NYC HANES 2004			

Total	1,840	0.74 (0.69–0.79)	4.35 (3.95–4.73)
Males	766	0.69 (0.63–0.76)	3.77 (3.50–4.36)
Females	1,074	0.77 (0.71–0.85)	4.76 (4.20–5.76)

NHANES 2003–2004^a^			

Total	1,529	0.50 (0.44–0.56)	3.33 (2.76–3.88)

Data from CDC (2009).
